# Impact of the 2014 International Society of Urological Pathology Grading System on Concept of High-Risk Prostate Cancer: Comparison of Long-Term Oncological Outcomes in Patients Undergoing Radical Prostatectomy

**DOI:** 10.3389/fonc.2019.01272

**Published:** 2019-11-19

**Authors:** Daimantas Milonas, Žilvinas Venclovas, Inga Gudinaviciene, Stasys Auskalnis, Kristina Zviniene, Nemira Jurkiene, Algidas Basevicius, Ausvydas Patasius, Mindaugas Jievaltas, Steven Joniau

**Affiliations:** ^1^Department of Urology, Lithuanian University of Health Sciences, Medical Academy, Kaunas, Lithuania; ^2^Department of Pathology, Lithuanian University of Health Sciences, Medical Academy, Kaunas, Lithuania; ^3^Department of Radiology, Lithuanian University of Health Sciences, Medical Academy, Kaunas, Lithuania; ^4^Department of Oncourology, National Institute of Cancer, Vilnius, Lithuania; ^5^Department of Urology, Leuven University Hospital, Leuven, Belgium

**Keywords:** high risk prostate cancer, ISUP 2014 grade groups, radical prostatectomy, clinical progression, survival

## Abstract

**Objective:** To investigate the relationship between the new International Society of Urological Pathology (ISUP) grading system, biochemical recurrence (BCR), clinical progression (CP) and cancer related death (CRD) after open radical prostatectomy (RP) and determine whether the 2014 ISUP grading system influences the concept of high-risk prostate cancer (HRPCa).

**Patients and Methods:** A total of 1,754 men who underwent RP from 2005 to 2017 were identified from a database at a single tertiary institution. Histopathology reports were reassessed according to the 2014 ISUP grading system. All preoperative, pathological, and clinical follow-up data were obtained. Univariable and multivariable Cox regression, Kaplan-Meier and log-rank analyses were performed.

**Results:** At a median (quartiles) follow-up of 83 (48–123) months, 446 men (25.4%) had BCR, 77 (4.4%) had CP and 39 (2.2%) died from cancer. Grade groups 1, 2, 3, 4, and 5 were detected in 404 (23%), 931 (53.1%), 200 (11.4%), 93 (5.3%), and 126 (7.2%), respectively. 10-year biochemical progression free survival difference between Grade group 3 and 4 was minor but significant (log-rank *p* = 0.045). There was no difference between Grade groups 3 and 4 comparing 10-year clinical progression free and 10-year cancer specific survival: *p* = 0.82 and *p* = 0.39, respectively. Group 5 had the worst survival rates in comparison with other groups (from *p* < 0.005 to *p* < 0.0001) in all survival analyses. Pathological stage (hazard ratio (HR) 2.6, *p* < 0.001), positive surgical margins (HR 2.2, *p* < 0.0001) and Grade group (HR 10.4, *p* < 0.0001) were independent predictors for BCR. Stage and Grade group were detected as independent predictors for CP–HR 6.0, *p* < 0.0001 and HR 35.6, *p* < 0.0001, respectively. Only Grade group 5 (HR 12.9, *p* = 0.001) and pT3b (HR 5.9, *p* = 0.001) independently predicted CRD.

**Conclusions:** The new ISUP 2014 grading system is the most significant independent predictor for BCR, CP, and CRD. Grade group 3 and 4 had similar long-term disease progression survival rates and could potentially be stratified in the same risk group. High-risk cancer associated only with group 5.

## Introduction

The Gleason score (GS) grading system is one of the strongest predictors for prostate cancer (PCa) outcomes and plays a significant role for choosing treatment modality. Since the 1960s when this grading system was developed by Donald Gleason (1), several modifications have been adopted. The International Society of Urological Pathology (ISUP) suggested the currently used GS system in 2005 (2). The division of GS into 6 vs. 7 vs. 8–10 together with corresponding grouping of the prostate specific antigen (PSA) and clinical stages into three groups—low, intermediate and high PCa risk groups,—are known as D'Amico classification (3) that has been adopted in clinical practice and has been widely used for prognostic and therapeutic purposes. The EAU PCa risk group classification, which is based on D'Amico criteria, is used until now (4). Current high-risk PCa definition included PSA >20 ng/ml or GS >7 or clinical stage (cT) ≥2c in localized, or cT3-4 or cN+ with any PSA and any GS for locally advanced PCa (5), and the GS is the most important parameter in these groupings. Recently, some studies have shown that scores 3+4 vs. 4+3, also 8 vs. 9–10 have a different prognosis (6–8). In 2013, based on the data presented by Pierorazio et al. from Johns Hopkins Hospital, a new grading system of five prognostic grade groups (GS ≤6—prognostic grade group 1, 3+4—group 2, 4+3—group 3, 8—group 4 and 9–10—group 5) was proposed (9). Very recently, in a large multi-institutional study, Epstein et al. have confirmed that the five-group ISUP 2014 grading system provides a more accurate grade stratification than the current ISUP 2005 model (10). Biochemical progression free survival (BPFS) was different in all five groups in patients after radical prostatectomy (RP) and radiation therapy (RT). One of the limitations in this study was the use of biochemical recurrence (BCR) as an end-point as opposed to clinical progression (CP) or cancer-related death (CRD). Grogan et al. confirm that the ISUP 2014 grading system is an independent predictor not only for BCR, but also for CP. Harrells' c-index for the ISUP 2014 grading was significantly higher compared to the ISUP 2005 grading system (11). Such recent, new clinical data influenced the addition of ISUP grades 4 and 5 to the definition of high-risk PCa suggested by EAU (12). The aim of the present study was to assess where the ISUP 2014 grading system reflects the recently proposed concept of high-risk PCa in a long-term follow-up cohort of men undergoing RP at a tertiary university hospital. The primary end-point was to assess the association between the ISUP 2014 grading and BPFS; the secondary end-points were to investigate the association between the new grading system and clinical progression free survival (CPFS) and cancer specific survival (CSS).

## Materials and Methods

Between 2005 and 2017, 2,255 men were treated by RP for clinically localized PCa at a single university hospital centre using similar surgical techniques. We identified 1,754 men with complete pathological and follow-up data. Clinical characteristics, such as PSA level, clinical stage (cT), and biopsy GS were reported before RP. Pathological parameters [pathological stage (pT), GS, surgical margin status (R0 vs. R1) and lymph nodes status N0 vs. N1] were collected after surgery. PSA testing after RP was performed every 3 months in the first year, biannually in the second and third year, and once a year thereafter. BCR was identified as a PSA value of >0.2 ng/ml in two consequent measurements. CP was identified upon skeletal or visceral lesions confirmations by bone scan, CT or MRI using RECIST criteria. Local and loco-regional recurrence was confirmed by histological investigation after surgery or biopsy. Pathological stage was assessed using 2002 TNM system and tumor grading was classified using the revised 2005 ISUP GS grading system (2). Histopathological investigation in the majority of cases was performed by one uropathologist. Adjuvant therapy (RT alone or RT + androgen deprivation therapy) was performed depending on the pathological characteristics of PCa within 6 months after RP and salvage therapy (RT alone or RT + androgen deprivation therapy or salvage lymph node dissection) was applied after detecting BCR. The university's ethical committee approved the prospective collection of the data and all patients signed a consent form provided before RP. According to the pathologist's reports, the 2005 Gleason grading model was reassessed to the five-group system: GS ≤6 (Grade group 1) vs. 3+4 (Grade group 2) vs. 4+3 (Grade group 3) vs. 8 (Grade group 4) vs. 9–10 (Grade group 5) according to the 2014 ISUP Consensus Conference (13). Mortality data were obtained from the National Cancer Registry and reassessed using the department database for clinical progression to ensure the accuracy of the cause of death. Time to BCR, CP, and CRD was defined as the time interval from surgery to the event. BPFS, CPFS and CSS were estimated using Kaplan-Meier analysis. The log-rank test was used to compare differences among groups. The impact of the new 2014 ISUP grouping on BCR, CP, and CRD was analyzed by using univariable and multivariable Cox regression in combination with other factors, such as preoperative PSA, pathological stage (pT2 vs. pT3a vs. pT3b and surgical margins status (R0 vs. R1). Variables that had *p* < 0.1 value in univariable analysis were included in the multivariable Cox proportional hazards model. A *p* < 0.05 value was considered as significant and all reported *p*-values were two-sided. Statistical analysis was performed using SPSS software version 23 (IBM).

## Results

The study cohort includes 1,745 men who underwent open RP for clinically localized PCa. Clinical and pathological characteristics of patients are shown in Table 1.

**Table 1:** Clinical and pathological characteristics of patients (*n* = 1,754).

**Characteristics**	
Age, yr-median (quartiles)	64 (59–68)
PSA, ng/ml-median (quartiles)	6.3 (4.7–9.8)
Clinical stage, *n* (%)	
cT1	481 (27.4)
cT2	995 (56.8)
cT3	278 (15.8)
**Biopsy Gleason score**, ***n*** **(%)**	
6	970 (55.3)
3+4	559 (31.9)
4+3	84 (4.8)
8	93 (5.3)
9–10	48 (2.7)
**Pathological stage**, ***n*** **(%)**	
pT2	1,046 (59.6)
pT3a	555 (31.6)
pT3b	153 (8.8)
**Pathological Gleason score**, ***n*** **(%)**	
6	404 (23.0)
3+4	931 (53.1)
4+3	200 (11.4)
8	93 (5.3)
9-10	126 (7.2)
Positive surgical margins (*n* = 16,77), *n* (%)	446 (32.5)
Positive lymph nodes (*n* = 618), *n* (%)	75 (12.1)

*PSA, prostate specific antigen*.

The median (quartiles) follow-up was 83 (48–123) months. BCR during the study period was observed in 446 (25.4%) men and CP—in 77 (4.4%) patients: local recurrence was detected in 7 (0.4%), loco-regional in 15 (0.9%) and distant lesions in 55 (3.1%) patients, respectively. There were 216 (12.3%) deaths during follow-up period and 39 (2.2%) documented as CRD.

**Figure 1:**
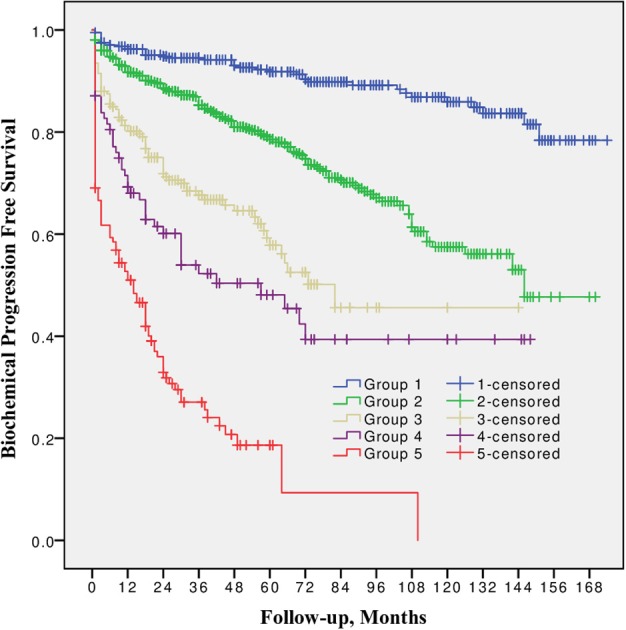
Biochemical progression free survival after radical prostatectomy stratified by 2014 International Society of Urological Pathology suggested Grade Group 1, 2, 3, 4, and 5.

10-year BPFS for Grade group 1, 2, 3, 4, and 5 was 85.9, 57.5, 45.6, 39.4 and 0.0%, respectively. The difference between all five groups (Figure 1) was significant (log-rank *p* from 0.045 to < 0.0001). The smallest difference was detected between groups 3 and 4 (*p* = 0.045).

**Figure 2:**
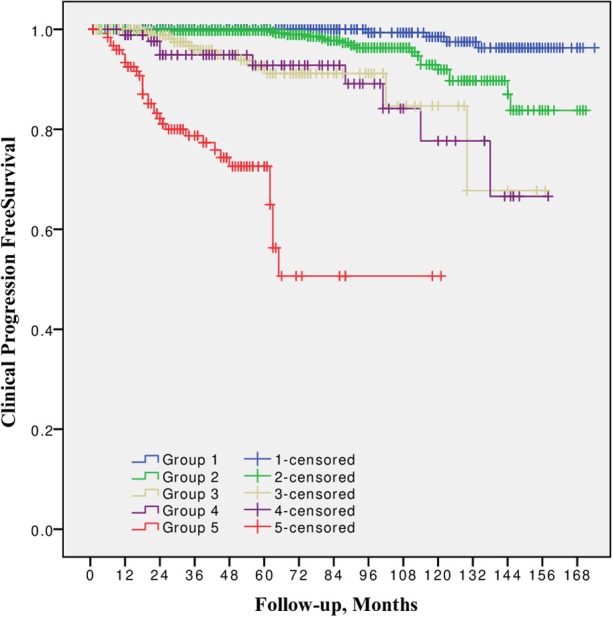
Clinical progression free survival after radical prostatectomy stratified by 2014 International Society of Urological Pathology suggested Grade Group 1, 2, 3, 4, and 5.

10-year CPFS was 98.5, 92.0, 84.7, 77.7, and 50.7% for Group 1, 2, 3, 4, and 5, respectively (Figure 2). The difference between all five groups was significant (*p* from 0.002 to < 0.0001), except between Grade group 3 vs. 4 (*p* = 0.8).

**Figure 3:**
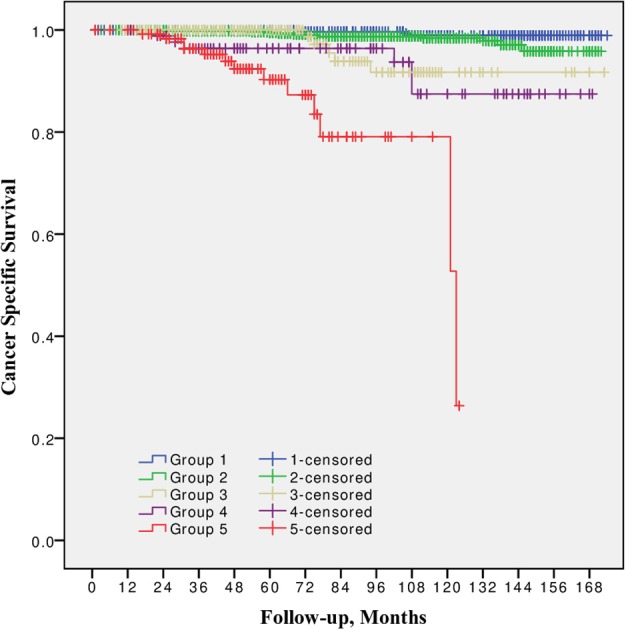
Cancer specific survival after radical prostatectomy stratified by 2014 International Society of Urological Pathology suggested Grade Group 1, 2, 3, 4, and 5.

10-year CSS was 98.9, 98.4, 91.7, 87.5, and 79.8% for Group 1, 2, 3, 4, and 5 respectively (Figure 3). The difference between Grade group 1 vs. 2, also between 3 vs. 4 was not significant (*p* = 0.09 and *p* = 0.4, respectively). Other pairwise comparison was significant (*p* from 0.02 to < 0.0001).

In univariable Cox regression analysis risk for BCR increased with a higher Grade group (*p* < 0.0001), a higher pT stage (*p* < 0.0001) and surgical margins status (*p* < 0.0001). Age and PSA were not significant predictors for BCR (Table 2). Higher risk of CP was associated with a higher Grade group (*p* from 0.01 to < 0.0001), a pathological stage (*p* < 0.0001) and positive surgical margins (*p* < 0.0001), but not with age and PSA (Table 3). Higher risk of CRD was associated with positive surgical margins (*p* < 0.0001), age (*p* = 0.003), stage after RP (*p* = 0.004 to *p* < 0.0001) and Grade group 3–5 (*p* = 0.002 to < 0.0001), but not with preoperative PSA (Table 4).

**Table 2:** Cox proportional hazards analysis of factors for prediction of biochemical recurrence after radical prostatectomy (*n* = 1,745).

**Parameter**	**Univariable analysis**		**Multivariable analysis**	
	**HR (95% CI)**	***p***	**HR (95% CI)**	***p***
Age (years)	1.0 (0.99–1.02)	0.28	–	–
Preoperative PSA (ng/ml)	1.0 (0.99–1.00)	0.31	–	–
Surgical margins (R0 vs. R1)	3.5 (2.85–4.20)	<0.0001	2.2 (1.77–2.69)	<0.0001
Pathological stage				
pT2				
pT3a	2.6 (2.14–3.29)	<0.0001	1.34 (1.05–1.71)	0.02
pT3b	8.4 (6.53–10.70)	<0.0001	2.2 (1.77–2.69)	<0.0001
Grade group				
1				
2	2.9 (2.09–4.16)	<0.0001	2.2 (1.53–3.16)	<0.0001
3	6.6 (4.43–9.74)	<0.0001	4.4 (2.88–6.76)	<0.0001
4	8.8 (5.73–13.49)	<0.0001	5.2 (3.29–8.35)	<0.0001
5	22.4 (15.21–32.87)	<0.0001	10.4 (6.67–16.15)	<0.0001

*PSA, prostate specific antigen*.

**Table 3:** Cox proportional hazards analysis of factors for prediction of clinical progression after radical prostatectomy (*n* = 1,745).

**Parameter**	**Univariable analysis**		**Multivariable analysis**	
	**HR (95% CI)**	***p***	**HR (95% CI)**	***p***
Age (years)	1.0 (0.99–1.07)	0.14	–	–
Preoperative PSA (ng/ml)	1.0 (0.99–1.00)	0.46	–	–
Surgical margins (R0 vs. R1)	3.7 (2.33–6.03)	<0.0001	1.4 (0.85–2.40)	0.11
**Pathological stage**				
pT2				
pT3a	4.7 (2.47–8.97)	<0.0001	2.3 (1.11–4.61)	0.02
pT3b	22.3 (11.04–41.53)	<0.0001	6.0 (2.91–12.54)	<0.0001
**Grade group**				
1				
2	4.1 (1.38-12.01)	0.01	2.5 (0.80-7.69)	0.1
3	16.8 (5.3–53.37)	<0.0001	7.1 (2.05–24.32)	0.002
4	16.1 (4.92–52.29)	<0.0001	7.6 (2.17–26.73)	0.002
5	125.3 (41.75–376.23)	<0.0001	35.6 (10.40–121.80)	<0.0001

*PSA, prostate specific antigen*.

**Table 4:** Cox proportional hazards analysis of factors for prediction of cancer related death after radical prostatectomy (*n* = 1,745).

**Parameter**	**Univariable analysis**		**Multivariable analysis**	
	**HR (95% CI)**	***p***	**HR (95% CI)**	***p***
Age (years)	1.1 (1.03–1.14)	0.003	1.1 (1.01–1.13)	0.013
Preoperative PSA (ng/ml)	1.0 (0.99–1.01)	0.8	–	–
Surgical margins (R0 vs. R1)	5.6 (2.69–11.50)	<0.0001	2.2 (0.99–4.95)	0.052
**Pathological stage**				
pT2				
pT3a	3.8 (1.55–9.3)	0.004	1.7 (0.63–4.76)	0.29
pT3b	24.3 (10.62–55.65)	<0.0001	5.9 (2.09–16.77)	0.001
**Grade group**				
1				
2	2.9 (0.80–10.19)	0.1	1.4 (0.37–5.57)	0.6
3	9.4 (2.20–40.39)	0.002	3.5 (0.72–16.93)	0.12
4	14.2 (3.54–56.95)	<0.0001	4.4 (0.95–20.26)	0.06
5	65.8 (17.66–245.51)	<0.0001	12.9 (2.78–60.08)	0.001

*PSA, prostate specific antigen*.

In multivariable analysis surgical margins status, pT and Grade group were detected as independent predictors (all *p* < 0.0001) for BCR (Table 2). The Grade group had the highest HR 10.4 compared to other parameters and could be used as the strongest predictor for PSA relapse. Stage and Grade group 3–5 had a significant impact on risk prediction also for CP (*p* = 0.02 to *p* < 0.0001) with the highest HR 35.6 in Grade group 5 (Table 3). Only Grade group 5 (HR 12.9, *p* = 0.001) and pT3b stage (HR 5.9, *p* = 0.001) were detected as independent predictors for CRD (Table 4).

In all univariable and multivariable Cox regression and log-rank analyses for BCR, CP and CRD Grade group 4 was much closer to Grade group 3 than to group 5. The HR difference between Grade group 4 and group 5 in various analyses was from two- to eight-fold, whereas between Grade group 4 and group 3 it was less than one-fold (Tables 2–4). The Kaplan-Meier survival curves were slightly different between Grade groups 3 and 4 (*p* = 0.045) analyzing BPFS and similar analyzing CPFS and CCS. Therefore, the difference between Grade groups 4 and 5 was significant in all survival curves (*p* = 0.005 to *p* < 0.0001), which shows different cancer aggressiveness in these groups (Figures 1–3).

## Discussion

The GS has been confirmed as one of the most powerful predictors of PCa progression in our previous studies (14, 15). Various GS have been grouped together based on the assumption that they could have a similar impact on cancer behavior (16–18). Therefore, the division of GS into three groups (≤6, 7, and 8–10) becomes most therapeutically relevant and used worldwide in various models (low, intermediate and high-risk D'Amico criteria) for the prognosis of PCa progression (3, 19). Until now, such grouping has been most popular and EAU guidelines recommended it for PCa risk stratification (5). However, recent publications have clearly demonstrated that GS 3+4 vs. 4+3 has different prognosis for biochemical and disease-free survival (6, 7). Also, some studies have shown that GS 9–10 has the worst prognosis and GS 8 is closer to 4+3 than to 9–10 (8). Cases with GS 9 and 10 are quite rare and this has been the main reason for putting them together with GS 8 for more powerful statistical conclusions. However, some very recent studies show different cancer behavior at GS 8 and 9–10 (20). This suggests that the currently used PCa stratification to low, intermediate and high-risk can harbor really very high aggressiveness of cancer with GS 9–10. Moreover, indications for surgical treatment of high-risk PCa has been changed during the last decade and cases with GS 9–10 after RP will becomes more and more often. Understanding about behavior such PCa becomes very relevant.

The new ISUP GS grouping to five groups was proposed in 2013: Grade Group 1 (GS ≤6)—only individual discrete well-formed glands; Grade Group 2 (GS 3+4 = 7)—predominantly well-formed glands with a lesser component of poorly formed/fused/cribriform glands; Grade Group 3 (GS 4+3 = 7)—predominantly poorly-formed/fused/cribriform glands with a lesser component of well-formed glands; Grade Group 4 (GS 8)—only poorly-formed/fused/cribriform glands or predominantly well-formed glands with a lesser component lacking glands or—predominantly lacking glands with a lesser component of well-formed glands; Grade Group 5 (GS 9–10)—lacks gland formation (or with necrosis) with or w/o poorly-formed/fused/cribriform glands (9). The effectiveness of the suggested model was confirmed in a larger than 25,000 men multi-institutional cohort by Epstein et al. The difference for 5-year BPFS varied among all groups and the detected HR was from two- to three-fold higher for each group comparing PSA relapse in patients not only after RP, but also after RT. This study clearly proves that the new GS grouping into five groups is a better prognosticator of BCR than the currently used three group model: Harrell's c-index was higher from 0.02 to 0.05 in biopsy, RP and RT cohorts (10). PSA relapse is not always associated with CP and CRD. Epstein et al. also pointed this out as a limitation of their study (10). Very recently, Grogan et al. have presented the results of patients who underwent RP (1991–1999) with median 15.25 years' follow-up. Histopathology reports were reviewed and assigned to Grade groups in line with the recommendations of the 2014 ISUP Consensus Conference. The authors have concluded that the ISUP 2014 grading system is a significant independent predictor of both BCR and CP, outperforming the 2005 ISUP modified Gleason system (11). The presented results of our study show the same tendencies: Grade group was the strongest independent predictor for BCR, CP and CRD in multivariable Cox analysis. There is no doubt that the ISUP 2014 grading system, referred to as Grade Group in the 2016 WHO Classification (21), will be used in the coming decades in clinical practice. Therefore, there is a need to know how it will influence the worldwide adapted stratification to low, intermediate and high-risk PCa.

The presented study results show some tendencies in cancer behavior, especially in that associated with the high-risk disease. Grade groups had different survival rates when analyzing earlier disease progression—BCR, but Grade group 4 curve was much closer to group 3 (10-year BPFS 39.4% vs. 45.6%, *p* = 0.045) than to group 5 (39.4 vs. 0.0%, *p* < 0.0001, Figure 1). In addition to this, clinical disease progression analysis revealed the closer survival rates between Grade groups 4 and 3 (10-year CPFS 77.7 vs. 84.7%, *p* = 0.8) than between groups 4 and 5 (77.7 vs. 50.7%, *p* < 0.0001, Figure 2). Finally, the 10-year CSS rates were different between Grade groups 4 and 5 (87.5 vs. 79.8%, *p* = 0.005) and similar between Grade groups 4 and 3 (87.5 vs. 91.7%, *p* = 0.4, Figure 3). The Cox regression proportional hazard ratio analysis confirmed such findings: in univariable and multivariable analysis, the HR comparing groups 4 and 3 was much closer than comparing groups 4 and 5 (differences from two- to eight-fold—Tables 2–4) and only group 5 was associated with CRD. The same tendencies in multivariable Cox regression for Grade groups 3, 4 and 5 have been shown by Grogan et al.: HRs 6.2 vs. 6.5 vs. 12.1 for BCR, and HRs 13.2 vs. 13.9 vs. 34.3 for CP, respectively. The authors did not show survival rate data, but the Kaplan-Meier curves presented by them are similar to those observed in our study (11).

Despite its benefits for better differentiation of PCa aggressiveness it is unclear how the 2014 ISUP suggested five Grade group scheme should be integrated into the currently used PCa risk models. If our findings are considered accurate, D'Amico criteria and other PCa risk stratification nomograms based on the three-grade GS model (GS 6/ISUP Grade 1—low-risk, GS 7/ISUP Grade 2/3—intermediate-risk and GS 8–10/ISUP Grade 4/5—high-risk PCa) covered very broad groups and should be reassessed and simplified. According to the results of the presented study, Grade group 5 associated with the highest risk for PCa progression and should be split from group 4. Grade groups 4 and 3 could be integrated into the same aggressiveness group because of their similar risk for progression. Grade group 1 and 2 shows very similar risk for disease progression and could be analyzed together. Using Grade groups 4 and 5 together for the definition of high-risk PCa poses a real risk because is masks the biggest aggressiveness of group 5. According to our results, Grade groups 1 and 2 could be integrate into the low-risk, Grade groups 3 and 4—into the intermediate and Grade group 5—into the high risk group.

The present study is not devoid of limitations: these are the relatively short follow-up, the absence of other treatment modality group and direct comparison of results and the relatively small number of cases with CP and CRD. Re-review of the pathology slides also could change the proportion between Grade groups. Relatively high, comparing to single surgeon series, positive surgical margins rate also could impact outcomes. On the other hand positive surgical margin was not confirmed as significant predictor of CP and CRD in multivariable Cox regression analysis. All these above mentioned limitations can influence the results and their interpretation.

The strength of the present study is prospectively collected data, standard evaluation of disease progression and treatment of BCR and pathological investigation by one experienced pathologist in the majority of cases. The end-point of this study was CP and CRD that are most important for cancer behavior analysis.

To our knowledge, there are very few studies that describe CP and CRD as end-point using the 2014 ISUP model after RP and there are no studies addressing high-risk PCa. More studies are needed to confirm our findings.

## Conclusions

The 2014 ISUP Grading model provides very accurate grade stratification and closely reflects cancer behavior and prognosis in patients after radical prostatectomy. Grade group 5 is associated with the highest risk for cancer progression and is significantly different from other groups. Grade group 3 and Grade group 4 have the same risk for PCa progression in long-term follow-up.

## Data Availability Statement

The datasets generated for this study are available on request to the corresponding author.

## Ethics Statement

The studies involving human participants were reviewed and approved by Kaunas Regional Biomedical Research Ethics Committee. The patients/participants provided their written informed consent to participate in this study.

## Author Contributions

DM: study design, statistical analysis, data collection, and manuscript writing. ŽV: statistical analysis, data collection, and manuscript revision. IG: data collection and manuscript writing. SA, KZ, NJ, AB and AP: data collection and manuscript revision. MJ: manuscript revision. SJ: study design and manuscript revision.

### Conflict of Interest

The authors declare that the research was conducted in the absence of any commercial or financial relationships that could be construed as a potential conflict of interest.
